# Determinants of Six-Minute Walk Distance in Idiopathic Pulmonary Fibrosis and Idiopathic Pleuroparenchymal Fibroelastosis

**DOI:** 10.3390/biomedicines10102556

**Published:** 2022-10-13

**Authors:** Naofumi Sato, Yuji Iwanami, Kento Ebihara, Keiko Nakao, Midori Miyagi, Yasuhiko Nakamura, Kazuma Kishi, Sakae Homma, Satoru Ebihara

**Affiliations:** 1 Department of Rehabilitation Medicine, Toho University Omori Medical Center, 6-11-1 Omori-nishi, Ota-ku, Tokyo 143-8541, Japan; 2 Department of Respiratory Medicine, Toho University School of Medicine, 6-11-1 Omori-nishi, Ota-ku, Tokyo 143-8541, Japan; 3 Department of Advanced and Integrated Interstitial Lung Diseases Research, Toho University School of Medicine, 6-11-1 Omori-nishi, Ota-ku, Tokyo 143-8541, Japan; 4 Department of Internal Medicine and Rehabilitation Science, Tohoku University Graduate School of Medicine, 1-1 Seiryo-machi, Aoba-ku, Sendai 980-5874, Japan

**Keywords:** idiopathic interstitial pneumonias, leanness, shortness of breath, severity of illness

## Abstract

Background: In idiopathic pulmonary fibrosis (IPF), 6-minute walking distance (6MWD) is an independent factor for mortality. Idiopathic pleuroparenchymal fibroelastosis (IPPFE) is a rare disease with physical features such as emaciation, but the relationship between IPPFE and 6MWD is unclear. In this study, we investigated the factors that cause a decrease in the percent of the predicted value of a 6-minute walk distance (%6MWD), including the disease entities, IPF and IPPFE. Methods: This study included 100 patients (IPF: 75 patients, IPPFE: 25 patients, age: 73.5 ± 7.2 years, sex: 73 males) who visited the rehabilitation department. Patients with a %6MWD ≥ 80% were assigned to the normal group (*n* = 54), and patients with a %6MWD < 80% were assigned to the decreased group (*n* = 46). The items showing a significant difference between groups were used as independent variables, after the consideration of multicollinearity, for a logistic analysis where %6MWD < 80% was used as the dependent variable. Results: The 6MWD results show that there was no significant difference between IPF and IPPFE in the absolute value of 6MWD and in the number of people with 6MWD ≥ 250 m, but when 6MWD was compared with %6MWD, the IPPFE group showed a significantly lower value than the IPF group (*p* = 0.013). Logistic regression analysis showed that only BMI (*p* = 0.032), GAP index (*p* = 0.043), and mMRC (*p* = 0.026) were factors that caused a decrease in %6MWD in 100 patients. Conclusion: The results suggest that leanness, shortness of breath and severity of illness are the most important factors that determine exercise tolerance, regardless of disease entity in IPF and IPPFE.

## 1. Introduction

Idiopathic interstitial pneumonias (IIPs) are pneumonias of unknown cause, in which the pulmonary interstitium is the primary site of inflammatory and fibrotic lesions. The main symptoms of IIPs are dry cough and dyspnea on exertion [1]. IIPs are classified into nine entities based on histopathological patterns, and each course and set of clinical features have disease specificity and diversity [2].

The 6-minute walk test (6MWT), a simple test of exercise tolerance, is used as an index for estimating motor function and disease severity and predicting prognosis in various chronic diseases [3]. Although 6-minute walking distance (6MWD) is an independent predictor of mortality in idiopathic pulmonary fibrosis (IPF) [4,5,6], which is the main entity of IIPs, it is unclear whether 6MWD is also an independent prognostic factor in the different disease entities of IIPs. Notably, 6MWD is affected by individual factors such as age, sex, and anthropometric data [3]. Older, shorter, and heavier people have shorter 6MWDs than their counterparts [7].

Based on idiopathic upper lobe localized pulmonary fibrosis, which was proposed by Amitani et al. in 1992 [8], idiopathic pleuroparenchymal fibroelastosis (IPPFE) was proposed by Frankel in 2004 as a new disease entity of IIPs [9]. Unlike IPF fibrotic lesions, predominantly found bilaterally in the lower lung fields, IPPFE fibrotic lesions are in the upper lung fields [9]. When the international classification of IIPs was revised in 2013, IPPFE, a rare interstitial pneumonia, was classified as one of the nine types of IIPs [2]. Patients with IPPFE are lean and present with a flat thorax, resulting in suppressed lung expansion during inspiration and a more rapid decrease in the forced vital capacity than in other IIPs [10,11]. Furthermore, although various opinions have been expressed regarding the progression of the disease, the prognosis of IPPFE is generally considered poor compared to other IPFs [12,13].

From the progressive decline in lung function and poor prognosis, it could be predicted that IPPFE reduces 6MWD more than other IIPs. However, previous reports comparing IPPFE and other IIPs in lung transplant candidates have shown no significant difference in 6MWD [14,15]. When 6MWD values were compared with the results for IPF in previous reports [4,5,6,16], the decrease in distance was mild, despite the marked decrease in forced vital capacity (FVC) [12]. One explanation for this contradiction is that leanness in IPPFE may increase walking distance. Therefore, it is better to consider anthropometric data when comparing 6MWD and to use the percent of predicted value of 6MWD (%6MWD), which is obtained by gender, height, and weight in individual subject [7].

In this study, we compared, for the first time, 6MWD as %6MWD between IPPFE and IPF, and the factors that cause a decrease in %6MWD in both IPF and IPPFE were examined to clarify the role of the disease entity as IPPFE.

## 2. Materials and Methods

### 2.1. Study Population and Selection Criteria

We recruited 273 consecutive patients who participated in the Toho University Interstitial Pneumonia Rehabilitation (TRIP) study from July 2014 to January 2020. The TRIP study was designed to clarify the long-term effect (2-year follow-up) of pulmonary rehabilitation in patients with interstitial lung disease (ILD), and it is an on-going project at the Toho University Omori Medical Center, Tokyo, Japan [17]. All of the patients participating in the TRIP study in the indicated period were recruited. When the patient was referred to the rehabilitation department, basic attributes such as respiratory function and physical function were assessed. Computed tomography (CT) imaging was performed within 2 weeks of these assessments. In this study, we used the data from the physical function evaluation performed at the first visit to the rehabilitation department.

The exclusion criteria for the TRIP study were active cancer, unstable cardiac disease, severe orthopedic or neurological disorder that limited exercise performance, previous participation in pulmonary rehabilitation (PR), and recent changes in medications, including antifibrotic drugs.

**Figure 1:**
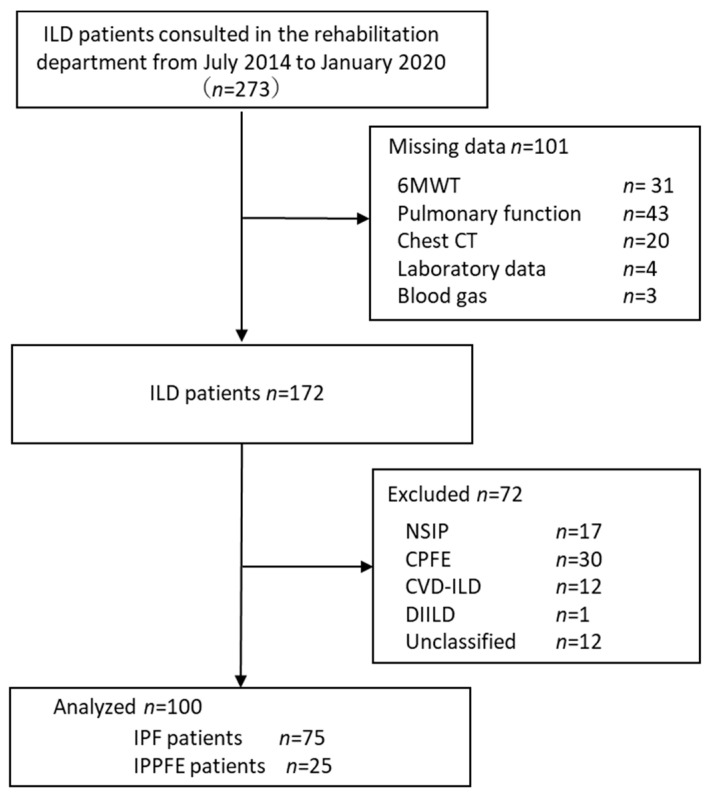
Flowchart of patient recruitment in this study. ILD, interstitial lung diseases; 6MWT, 6-minute walk test; chest CT, computed tomography; NSIP, nonspecific interstitial pneumonia; CPFE, combined pulmonary fibrosis and emphysema; CVD-ILD, collagen vascular disease–interstitial lung disease: DIILD, drug-induced interstitial lung disease; IPF, idiopathic pulmonary fibrosis; IPPFE, idiopathic pleuroparenchymal fibroelastosis.

In this study, 75 IPF patients and 25 IPPFE patients were included in the analysis, excluding those with missing data or other diseases entities (Figure 1). 

IPPFE was diagnosed both radiologically and physiologically. Probable IPPFE was diagnosed based on the criteria for diagnosis of IPPFE without surgical lung biopsy, as proposed by the Study Group on Diffuse Pulmonary Disorders, Scientific Research/Research on Intractable Diseases in Japan [18]. IPF was diagnosed using the American Thoracic Society/European Respiratory Society (ATS/ERS) statement at a multidisciplinary discussion (MDD) [19].

All study participants provided written informed consent. This study was approved by the Ethics Committee of Toho University Omori Medical Center (approval number 27-82).

### 2.2. Clinical Endpoints of the Original Study

We performed pulmonary function tests, including spirometry, total lung capacity (TLC), residual volume (RV), diffusing capacity of the lung for carbon monoxide (DLco), and blood gas analysis. Forced vital capacity (FVC), forced expiratory volume in 1.0 s (FEV_1_), FEV_1_/FVC ratio, TLC, RV, and DLco were measured using CHESTAC-8900 (DN) (CHEST, Tokyo, Japan). Each measured value was divided by the predicted value of each item, and the percentage of the predicted value (%pred) was calculated.

For severity, Gender–Age–Physiology (GAP) index [20], and evaluate dyspnea, we used the modified Medical Research Council (mMRC) [21].

Physical function was evaluated by grip strength, lower limb muscle strength, and 6MWT. The 6MWT was performed according to the published international guidelines [22]. All patients underwent at least one 6MWT prior to entry into the study to exclude any training effect on 6MWD. As a feature of 6MWT, individual body shape, such as weight and height, are independent factors of walking distance [8]. In this study, we used %6MWD, which is the ratio of the predicted value to the measured value, to evaluate the deviation from the abilities that people of the same age and size should have. The prediction formula proposed by Enright et al. [7] was used.

We quantitatively evaluated the cross-sectional area of the erector spinae muscle (ESM_CSA_) and the pectoralis major muscle (PM_CSA_) using CT images as indicators of skeletal muscle [23]. The analysis was performed using the software ImageJ, which is provided free of charge by the National Institutes of Health (https://imagej.net, accessed on 27 April 2020).

### 2.3. Grouping

To investigate the factors that cause a decrease in %6MWD, including both IPF and IPPFE, 80% of 6MWD was used as the cut-off value based on a previous study on healthy elderly subjects [24] and chronic obstructive pulmonary disease (COPD) [25]. Patients with ≥80% were assigned to the “normal group,” and patients with <80% were assigned to the “decline group.” In total, 54 patients (IPF: 46, IPPFE: 8) were assigned to the “normal group”, and 46 patients (IPF: 29, IPPFE: 17) were assigned to the “decline group.”

### 2.4. Statistical Analysis

The Mann–Whitney U test was used to compare ordinal and interval scales, and the χ2 test was used to compare nominal scales between disease types and groups for each endpoint. To examine whether %6MWD was relevant, binomial logistic regression analysis was performed using the items with *p* < 0.05, as independent variables in grouping by %6MWD. Multicollinearity was checked by Variance Inflation Factor. The statistical software used was SPSS version 17 (SPSS Inc., Chicago, IL, USA). The significance level was set at 5%.

## 3. Results

Comparison of IPF and IPPFE is presented in Table 1. Body mass index (BMI), mMRC, FVC, %FVC, FEV_1_, %FEV_1_, TLC, partial pressure of arterial carbon dioxide (PaCO_2_), muscle cross-sectional area, and muscle strength were significantly smaller in IPPFE than in IPF. FEV1/FVC and RV/TLC were significantly greater in patients with IPPFE than in those with IPF. There were no significant differences in age, gender, severity (GAP index), %TLC, %DLco, pH, partial pressure of oxygen in arterial blood (PaO_2_), and serum interstitial pneumonia markers (such as Krebs von den Lungen-6 [KL-6], surfactant protein A [SP-A], and surfactant protein D [SP-D]) between the groups.

The 6MWD results show that there was no significant difference between IPF and IPPFE in the absolute value of 6MWD and in the number of people with 6MWD ≥ 250 m, but when 6MWD was compared with %6MWD, the IPPFE group showed a significantly lower value than the IPF group (*p* = 0.013).

To elucidate whether the decrease in %6MWD was specific for IPPFE, logistic multivariate analysis was performed to identify the factors for %6MWD decline in patients with IPF and IPPFE. Patients with %6MWD ≥ 80% were assigned to the normal group, and patients with a %6MWD < 80% were assigned to the decreased group (Table 2). All items in Table 2, except age, RV, pH, PaCO_2_, and serum interstitial pneumonia markers, show significantly lower values in the decline group than in the normal group, suggesting that they were candidates for the independent variables in the logistic analysis setting, and whether %6MWD declined to <80% was the dependent variable.

The absolute value of the correlation coefficient between the GAP index and %FVC was 0.725, and that between the GAP Index and %DLco was 0.536. Since the GAP index includes both FVC% and DLco, the physiology component of this index, which is a well-established severity score for IPF [20], is also based on %FVC and %DLco. The GAP index was included as the independent factor in the binomial logistic regression analysis rather than %FVC and/or %DLco.

Finally, we conducted a binomial logistic analysis using the forced entry method with the following independent variables: disease entity, gender, GAP index, BMI, mMRC, %FEV_1_, %TLC, PaO_2_, grip strength, and cross-sectional area of pectoral and erector spinae muscles. As a result (Table 3), three factors were extracted as independent factors—BMI (*p* = 0.032, odds ratio 0.745, 95% confidence interval: 0.570–0.974), GAP index (*p* = 0.043, odds ratio 3.483, 95% confidence interval: 1.038–11.684), and mMRC (*p* = 0.026, odds ratio 2.131, 95% confidence interval: 1.097–4.139). The difference in disease entity between IPF and IPPFE was not extracted as an independent factor for %6MWD decline.

## 4. Discussion

In this study, although there was no significant difference in the absolute distance of the 6MWT, %6MWT was significantly smaller in IPPFE than in IPF. However, the results of logistic multivariate analysis of IPF and IPPFE together suggested that the difference was not related to the disease entity, i.e., IPF or IPPFE, but to BMI, dyspnea status, and severity of the disease evaluated by the GAP Index.

One may wonder why BMI was a factor limiting the performance of 6MWT after the correction for weight and height. BMI is a measure of a person’s leanness or corpulence, calculated as weight in kilograms (kg) divided by height in meters squared (m^2^). It is widely used as a general indicator of whether a person has a healthy body weight for height. However, the prediction equation of 6MWD was generated as a gender-specific linear regression of age, weight, and height, not directly reflecting leanness or corpulence [7]. Therefore, if the effect of leanness or corpulence is strong, BMI may be extracted as a distinguishing factor.

In patients with IPF, both BMI and fat-free mass index were positively correlated with the absolute values of 6MWD, suggesting that the loss of skeletal muscle has a negative effect on walking distance [26]. The negative effect of low BMI on 6MWT was significant even after adjusting for height and weight in subjects with IPF, as well as those with IPPFE in our study. Guler et al. [27] showed that the 4 m gait speed in patients with interstitial lung diseases was positively correlated with upper limb muscle, but not with lower limb muscle and negatively correlated with percent body fat. These results suggest that upper body skeletal muscle mass plays a pivotal role in the walking distance in patients with interstitial lung diseases.

IPPFE experiences weight loss even before the onset of the disease, and a deformity of the chest wall called “flat chest” occurs [28].

Therefore, the smaller %6MWD in IPPFE may be attributed to the leanness in IPPFE. Since leanness is usually accompanied by a smaller body weight, it may contribute to the preservation of walking capacity, resulting in a mild decrease in the absolute value of walking distance despite the markedly decreased FVC in IPPFE. Therefore, after the correction of body weight, the difference in 6MWT between IPF and IPPFE became apparent.

In contrast to interstitial lung diseases, BMI is negatively correlated with the absolute value of 6MWD in patients with COPD [29,30]. However, similar to interstitial lung diseases, fat-free mass index and loss of skeletal muscle are related to decreased walking distance [31,32]. Recently, sarcopenic obesity has also been reported to be associated with a decrease in 6MWD in patients with COPD [33]. Further longitudinal body composition studies in patients with COPD and interstitial lung disease are needed.

We used 80% as the cut-off value for %6MWD in grouping. Although there is no clear consensus on the cut-off value for %6MWD, Troosters et al. [24] reported that %6MWD < 82% can be considered abnormal in healthy elderly subjects. Moreover, COPD patients with 6MWD > 80% showed better global initiative for chronic obstructive lung disease (GOLD) classification (B-C-D or II-III-IV) [34] and a significantly smaller risk of exacerbation [25]. Therefore, we believe that the cut-off point of 80% for %6MWD is reasonable in our subjects as well. Previous studies have reported lung function such as VC, FVC, DLco, breathlessness, and health-related quality of life as factors contributing to the decrease in 6MWD in IPF [5,35]. In our study, the factors contributing to the decrease in %6MWD were BMI; mMRC, a measure of breathlessness; and GAP index, a measure of severity that includes %FVC and %DLco in lung function. Thus, the causes of decreased exercise performance during a 6-minute walk seem to be common between IPF and IPPFE, suggesting a common strategy to improve exercise performance, which is a prognostic factor in both disease entities.

One of the limitations of this study is that IPPFE did not use surgical lung specimens but employed a combination of both imaging criteria and physiological criteria using RV/TLC and BMI [19].

Furthermore, a subgroup of IPPFE patients with lower lobe usual interstitial pneumonia (UIP) showed a more rapid progress in the clinical course than those without lower lobe UIP in previous studies [36,37]. Therefore, it is important to examine the difference in the effect on physical fitness, such as 6MWD, depending on the presence or absence of UIP in the lower lobe, which was difficult in this study due to the problem of sample size.

In addition, we did not investigate the effects of the Flat Chest Index and 6MWD associated with IPPFE respiratory function [38].

Another possible limitation of the study is the limited sample size, which may compromise our ability to detect significant differences.

## 5. Conclusions

Our results suggest that, in a mixed population of IPF and IPPFE patients, leanness, shortness of breath, and severity of illness are the most important factors that determine exercise tolerance regardless of disease entity, i.e., IPF or IPPFE.

## Figures and Tables

**Table 1:** Comparisons between IPF and IPPFE.

	IPF (*n* = 75)	IPPFE (*n* = 25)	*p* Value
Age (years)	73.3 ± 7.6	73.4 ± 6.1	0.511 ^†^
Gender (male/female)	58/17	15/10	0.093 ^‡^
BMI (kg/m^2^)	23.5 ± 3.1	17.6 ± 2.3	<0.001 ^†^
mMRC (0/1/2/3/4)	19/29/16/8/3	3/5/10/4/3	0.010 ^†^
Severity			
GAP Index (Ⅰ/Ⅱ/Ⅲ)	35/30/10	8/10/7	0.097 ^†^
Pulmonary function			
FVC (L)	2.3 ± 0.7	1.6 ± 0.5	<0.001 ^†^
FVC (%predicted)	76.6 ± 19.1	58.2 ± 15.6	<0.001 ^†^
FEV_1_ (L)	1.9 ± 0.5	1.4 ± 0.5	0.001 ^†^
FEV_1_ (%predicted)	95.0 ± 24.2	78.0 ± 19.9	0.006 ^†^
FEV_1_/FVC (%)	89.9 ± 16.4	96.6 ± 17.5	0.005 ^†^
TLC (L)	3.6 ± 1.0	3.2 ± 0.9	0.044 ^†^
TLC (%predicted)	74.7 ± 16.5	70.0 ± 15.8	0.209 ^†^
RV (L)	1.3 ± 0.4	1.6 ± 0.7	0.430 ^†^
RV (%predicted)	79.7 ± 22.9	98.6 ± 33.4	0.020 ^†^
RV/TLC (%)	37.6 ± 7.5	48.9 ± 8.9	<0.001 ^†^
RV/TLC (%predicted)	100.6 ± 22.3	131.2 ± 28.3	<0.001 ^†^
DLco (%predicted)	60.6 ± 16.4	61.7 ± 17.0	0.786 ^†^
pH	7.4 ± 4.2	7.4 ± 0.0	0.693 ^†^
PaO_2_ (mmHg)	86.5.7 ± 13.9	86.5 ± 13.6	0.705 ^†^
PaCO_2_ (mmHg)	40.7 ± 4.2	45.5 ± 6.4	<0.001 ^†^
Serum IP marker			
KL-6 (U/mL)	978.8 ± 663.0	964.5 ± 299.9	0.159 ^†^
SP-A (ng/mL)	65.5 ± 31.8	59.1 ± 37.5	0.128 ^†^
SP-D (ng/mL)	261.7 ± 210.8	267.1 ± 201.2	0.982 ^†^
Physical assessment			
6MWD (m)	395.1 ± 106.9	373.4 ± 103.2	0.627 ^†^
≥250 m {No. (%)}	6 (8)	2 (8)	0.682 ^‡^
%6MWD (%predicted)	84.9 ± 22.5	72.1 ± 23.0	0.013 ^†^
Quadriceps force (Nm/kg)	1.3 ± 0.4	1.2 ± 0.5	0.471 ^†^
Handgrip strength (kg)	27.3 ± 7.5	22.4 ± 7.1	0.007 ^†^
Skeletal muscle assessment			
PM_CSA_ (cm^2^)	34.8 ± 8.4	24.9 ± 7.2	<0.001 ^†^
ESM_CSA_ (cm^2^)	29.6 ± 6.8	22.6 ± 6.4	<0.001 ^†^

The values are mean ± SD. ^†^ Data analyzed by Mann–Whitney U-test; ^‡^ Data analyzed by chi-square test. BMI, body mass index; mMRC, modified British Medical Research Council questionnaire; GAP, Gender–Age–Physiology; FVC, forced vital capacity; FEV_1_, forced expiratory volume in 1.0 s; RV, residual volume; TLC, total lung capacity; DLco, diffusing capacity of the lung for carbon monoxide; 6MWD, 6-minute walk distance; PaO_2_, partial pressure of arterial oxygen; PaCO_2_, partial pressure of arterial carbon dioxide; IP, interstitial pneumonia; KL-6, Krebs von den Lungen-6; SP-A, surfactant protein A; SP-D, surfactant protein D; PM_CSA_, cross-sectional area of pectoralis major muscle; ESM_CSA_, cross-sectional area of erector spinae muscle.

**Table 2:** Comparisons between groups by %6MWD (≥ 80% or <80%).

	Normal Group (*n* = 54)	Decline Group (*n* = 46)	*p* Value
IPF/IPPFE (*n*)	46/8	29/17	0.011 ^‡^
Age (years)	72.7 ± 7.5	74.5 ± 6.7	0.163 ^†^
Sex (male/female)	45/9	28/18	0.008 ^‡^
BMI (kg/m^2^)	23.4 ± 3.7	20.5 ± 3.6	0.002 ^†^
mMRC (0/1/2/3/4)	16/26/11/1/0	6/8/15/11/6	<0.001 ^†^
Severity			
GAP Index (I/II/III)	33/19/2	10/21/15	<0.001 ^†^
Pulmonary function			
FVC (L)	2.4 ± 0.7	1.8 ± 0.6	<0.001 ^†^
FVC (%predicted)	79.1 ± 17.7	63.7 ± 19.6	<0.001 ^†^
FEV_1_ (L)	2.0 ± 0.5	1.6 ± 0.5	<0.001 ^†^
FEV_1_ (%predicted)	96.5 ± 20.1	84.0 ± 27.2	0.002 ^†^
FEV_1_/FVC (%)	88.9 ± 16.2	94.7 ± 17.5	0.011 ^†^
TLC (L)	3.9 ± 0.9	3.2 ± 0.9	*p* < 0.001 ^†^
TLC (%predicted)	77.5 ± 15.3	68.8 ± 16.5	0.005 ^†^
RV (L)	1.4 ± 0.4	1.4 ± 0.6	0.181 ^†^
RV (%predicted)	86.3 ± 25.9	82.5 ± 28.5	0.209 ^†^
RV/TLC (%)	37.5 ± 15.3	44.2 ± 9.2	0.001 ^†^
%RV/TLC (%predicted)	99.8 ± 24.3	118.5 ± 27.5	0.001 ^†^
DLco (%predicted)	65.9 ± 14.9	54.5 ± 16.8	0.001 ^†^
pH	7.4 ± 0.0	7.4 ± 0.0	0.157 ^†^
PaO_2_ (mmHg)	89.0 ± 12.9	82.4 ± 14.4	0.036 ^†^
PaCO_2_ (mmHg)	41.3 ± 3.9	42.6 ± 6.6	0.352 ^†^
Serum IP marker			
KL-6 (U/mL)	888.0 ± 651.4	931.6 ± 453.7	0.201 ^†^
SP-A (ng/mL)	60.2 ± 31.6	68.8.1 ± 36.3	0.170 ^†^
SP-D (ng/mL)	247.0 ± 192.6	286.1 ± 233.4	0.197 ^†^
Physical assessment			
Quadriceps force (Nm/kg)	1.3 ± 0.4	1.2 ± 0.4	0.054 ^†^
Handgrip strength (kg)	29.1 ± 6.9	22.4 ± 6.9	<0.001 ^†^
Skeletal muscle assessment			
PM_CSA_ (cm^2^)	36.1 ± 8.8	27.9 ± 7.7	<0.001 ^†^

The values are mean ± SD, ^†^ Data analyzed by Mann–Whitney U-test, ^‡^ Data analyzed by chi-square test. BMI, body mass index; mMRC, modified British Medical Research Council questionnaire; GAP, Gender–Age–Physiology; FVC, forced vital capacity; FEV_1_, forced expiratory volume in 1.0 s; RV, residual volume; TLC, total lung capacity; total lung capacity ratio; DLco, diffusing capacity of the lung for carbon monoxide; 6MWD, 6-minute walk distance; PaO_2_, partial pressure of arterial oxygen; PaCO_2_, partial pressure of arterial carbon dioxide; IP, interstitial pneumonia; KL-6, Krebs von den Lungen-6; SP-A, surfactant protein A; SP-D, surfactant protein D; PM_CSA_, cross-sectional area of pectoralis major muscle; ESM_CSA_, cross-sectional area of erector spinae muscle.

**Table 3:** Binomial logistic analysis with %6MWD (<80%) as the dependent variable.

	Odds Ratio	95% CI	*p* Value
Disease type (IPF/IPPFE)	0.383	0.650–2.444	0.288
Sex (male/female)	2.103	0.238–18.535	0.503
BMI (kg/m^2^)	0.745	0.570–0.974	0.032
mMRC (0/1/2/3/4)	2.131	1.097–4.139	0.026
Severity			
GAP Index (I/II/III)	3.483	1.038–11.684	0.043
Pulmonary function			
FEV_1_ (%predicted)	1.009	0.961–1.060	0.708
TLC (%predicted)	1.002	0.941–1.060	0.962
RV/TLC (%predicted)	0.992	0.951–1.034	0.700
PaO_2_ (mmHg)	0.988	0.942–1.036	0.619
Physical assessment			
Handgrip strength (kg)	0.911	0.803–1.034	0.149
Skeletal muscle assessment			
PM_CSA_ (cm^2^)	0.951	0.846–1.070	0.407
ESM_CSA_ (cm^2^)	1.065	0.937–1.210	0.338

OR; odds ratio, 95% CI; 95% confidence interval. Independent variables: BMI, body mass index; mMRC, modified British Medical Research Council questionnaire; GAP, Gender–Age–Physiology; FEV_1_, forced expiratory volume in 1.0 s; RV, residual volume; TLC, total lung capacity; PaO_2_, partial pressure of arterial oxygen; PM_CSA_, cross-sectional area of pectoralis major muscle; ESM_CSA_, cross-sectional area of erector spinae muscle.

## Data Availability

The data presented in this study are available on request from the corresponding authors.
